# Deleterious coding variants in multi-case families with non-syndromic cleft lip and/or palate phenotypes

**DOI:** 10.1038/srep30457

**Published:** 2016-07-26

**Authors:** Reuben J. Pengelly, Liliana Arias, Julio Martínez, Rosanna Upstill-Goddard, Eleanor G. Seaby, Jane Gibson, Sarah Ennis, Andrew Collins, Ignacio Briceño

**Affiliations:** 1 Genetic Epidemiology and Genomic Informatics, Faculty of Medicine, University of Southampton, Southampton, UK; 2 Department of Biomedical Sciences, Medical School, Universidad de La Sabana, Bogota, Colombia; 3 Centre for Biological Sciences, Faculty of Natural & Environmental Sciences, University of Southampton, Southampton, UK

## Abstract

Nonsyndromic Cleft Lip and/or Palate (NSCLP) is regarded as a multifactorial condition in which clefting is an isolated phenotype, distinguished from the largely monogenic, syndromic forms which include clefts among a spectrum of phenotypes. Nonsyndromic clefting has been shown to arise through complex interactions between genetic and environmental factors. However, there is increasing evidence that the broad NSCLP classification may include a proportion of cases showing familial patterns of inheritance and contain highly penetrant deleterious variation in specific genes. Through exome sequencing of multi-case families ascertained in Bogota, Colombia, we identify 28 non-synonymous single nucleotide variants that are considered damaging by at least one predictive score. We discuss the functional impact of candidate variants identified. In one family we find a coding variant in the *MSX1* gene which is predicted damaging by multiple scores. This variant is in exon 2, a highly conserved region of the gene. Previous sequencing has suggested that mutations in *MSX1* may account for ~2% of NSCLP. Our analysis further supports evidence that a proportion of NSCLP cases arise through monogenic coding mutations, though further work is required to unravel the complex interplay of genetics and environment involved in facial clefting.

Cleft lip and/or palate (CLP) phenotypes are among the most frequent birth defects occurring at rates of 1/500–1/2500 births^1^. A proportion of cases present with syndromic disease (CLP in addition to a spectrum of additional phenotypes) mostly caused by rare mutations in single genes that often show Mendelian patterns of inheritance. However up to 70% of cases show phenotypes lacking any additional cognitive or craniofacial abnormalities, referred to as nonsyndromic cleft lip and/or palate (NSCLP). Such phenotypes are regarded as genetically complex arising through the interplay of numerous genetic and environmental factors. Increased understanding of the underlying aetiology of NSCLP phenotypes (both genetic and environmental) is needed to ultimately develop strategies for prevention, and improve treatment and prognosis. NSCLP has a significant genetic basis, for example, the first degree relatives of affected individuals have a 30–40 fold elevated risk and phenotype concordance for monozygotic (MZ) twins is 40–60%, compared to 5% for di-zygotic twins^1^. Genetic studies including linkage analysis, genome-wide association (GWAS), and GWAS-based meta-analysis, have yielded reproducible evidence for the involvement of several genes and gene regions. Collins *et al.*^2^, listed 16 genes and gene regions which have been firmly implicated in NSCLP through linkage and association analysis. Several of these are broad regions where the underlying causal variant(s) have yet to be pinpointed, however, polymorphisms in genes such as *IRF6* are strongly associated with NSCLP^3^ and more minor roles have been established for *MSX1*^4^,^5^, *PVRL1*, *FGFR2*, *PAX7*, *NOG* and *SPRY2* among others^6^.

Exome sequencing presents opportunities to identify rare coding variation that may contribute to risk of NSCLP phenotypes. If NSCLP is entirely multifactorial, the contribution of rarer variants may be largely polygenic and mediated by numerous variants of very small individual effect. In this case, causal genes may only be detectible through the analysis of large numbers of patients using, for example, burden tests^7^. However, there is growing evidence for involvement of rare variants of larger effect in NSCLP including, for example, truncating mutations in the *ARHGAP29* gene^8^ and mutations in the *IRF6* gene, which is also known to contain mutations involved in malformation syndromes that include CLP such as Van der Woude^9^. We consider here a number of NSCLP families with multiple affected individuals and undertake exome sequencing to investigate the contribution of rare variants in genes previously associated with any form of clefting phenotype.

## Materials and Methods

**Figure 1:**
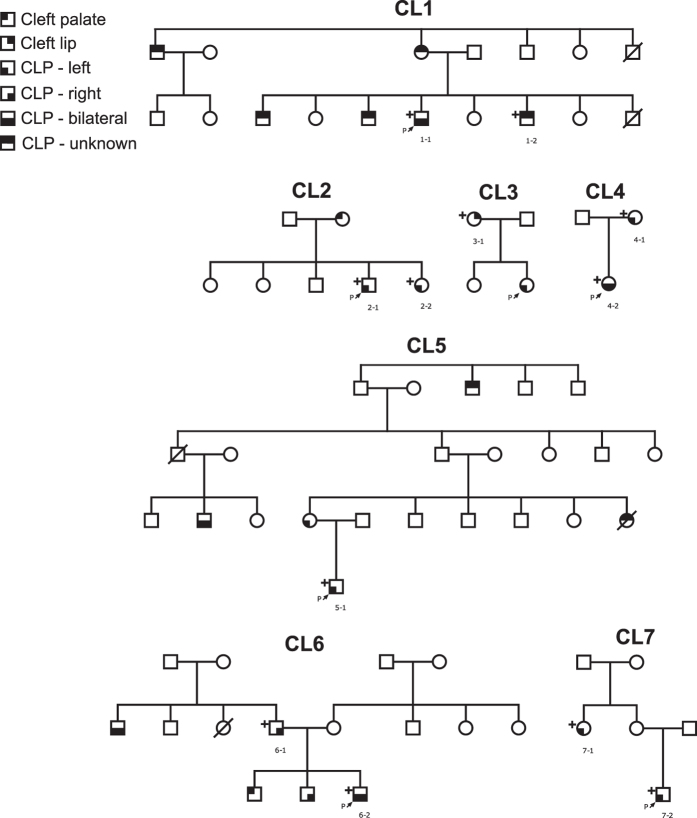
Pedigrees of families analysed. + symbol indicates that the individual has been exome sequenced (sequenced cases: two families with one family member; two families with parent and offspring; two families with sib pair; one family with avuncular pair).

Exome sequences of twelve individuals from seven multi-case families (CL1-CL7) with NSCLP phenotypes were obtained. All experimental protocols were approved by the Research Ethics Committee at the Universidad de La Sabana, Bogota; informed consent was obtained for all participants and research was conducted in accordance with the Declaration of Helsinki. Families included between two and six individuals with isolated NSCLP (Fig. 1). Most individuals have unilateral CLP but several individuals have the more severe bilateral phenotype.

DNA samples were extracted from blood collected at Operation Smile, Bogota, Colombia and exomes were captured using the Agilent SureSelect v5 (51 Mb) kit and sequenced on a HiSeq 2000. Read depth coverage statistics for all 12 exome sequences are given in Supplementary Table 1, and indicate ~85–97% coverage of exon targets at >20 fold depth across all samples. Orthogonal genotyping was performed for a panel of 24 SNPs to validate sample identity after processing^10^.

To understand the spectrum of potentially damaging variation, we considered the list of 865 genes previously implicated in any form of CLP phenotype presented by Pengelly *et al.*^11^ (Supplementary Table 2). Examining rare variation in genes in this comprehensive list enables evaluation of whether known CLP genes contain variation which may underlie more familial forms of NSCLP. Furthermore, because each exome contains a very large number of putatively damaging variants including those completely unrelated to the clefting phenotypes (including potential incidental findings), this strategy focussing only on genes previously implicated in any form of clefting is a practical route to identifying causal variation in these families. The list is derived in part (363 genes out of the 865) from the professional Human Gene Mutation Database^12^, using search terms related to clefts and clefting syndromes. The remaining genes in the list were included after corresponding interrogation of OMIM^13^, and a small number of additional CLP-related genes from the review by Collins *et al.*^2^.

**Figure 2:**
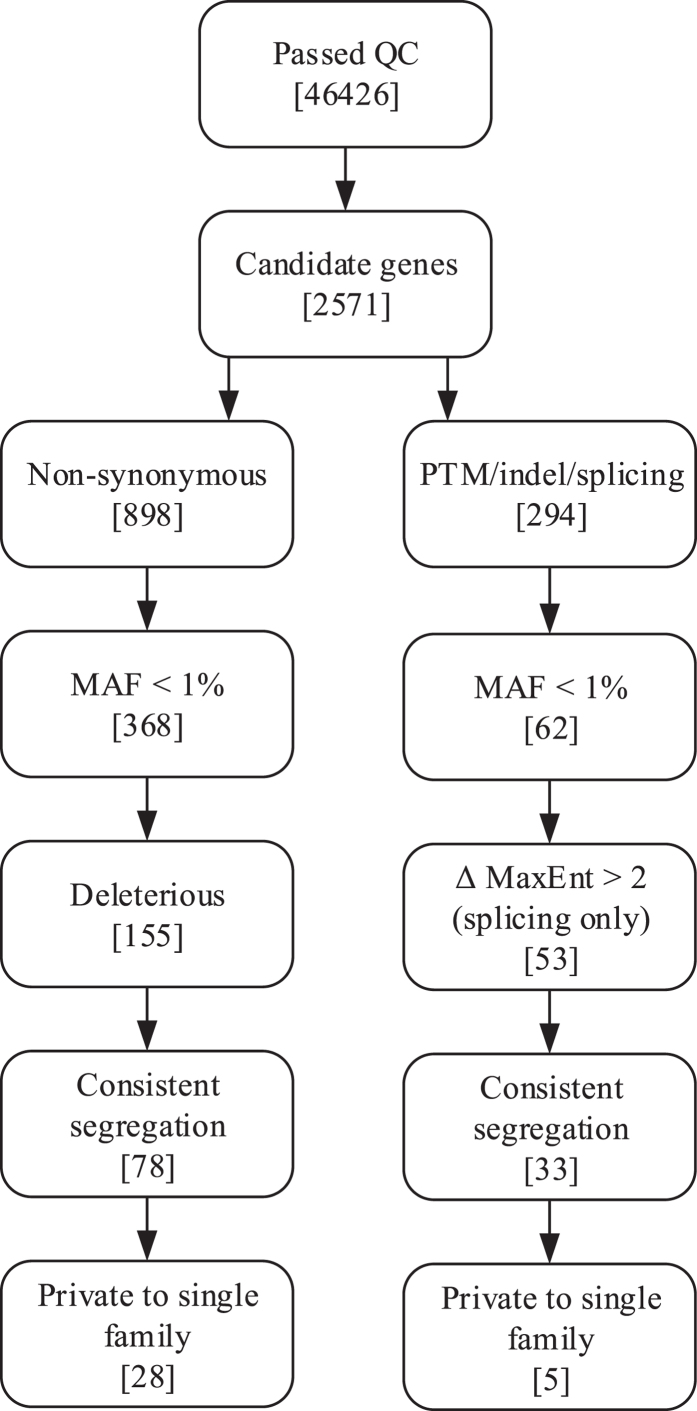
Variant filtering process. Variants identified in patients were filtered as described in methods. Variant attrition at each step is shown here, with variants remaining after sequential filtering detailed in square brackets.

We filtered the lists of variants (Fig. 2) found in the exome sequences to identify all non-synonymous (NS), stopgain, stoploss, splicing and indel variants in genes from this list. Following Pengelly *et al.*^11^, for NS variants we used the scaled predictive scores from dbNSFP v2^14^ and considered only variants classed as deleterious or damaging by at least one of the following predictive metrics: PhyloP, SIFT, Polyphen2, LRT, MutationTaster and GERP++. Grantham scores were also assigned to all NS substitutions. All variants were annotated with the minor allele frequency (MAF) from the ExAC database^15^, combined CADD and Logit scores for deleteriousness, along with a combined overall rank developed from PhylopP, GERP++, CADD and Logit scores based on the summed ranks across all four scores such that a variant with overall rank 1 is predicted as most deleterious. For intronic variants within 10 bp of the exon we utilised MaxEntScan, based upon quantifying deviation from the expected splicing consensus sequence motif, to evaluate the likelihood of this variant affecting splicing, using a cutoff of a differential score of 3^16^.

We excluded variants found in homopolymer/repeat regions that can arise through misalignment between the sequenced reads and reference sequence. Any variants with read depth of <10 or in genes considered to be ‘highly mutable’^17^ were removed from further consideration. We included all variants not previously listed in the following databases: dbSNP 135^18^, 1000 genomes^19^, the exome variant server^20^ and our in-house database of ~300 exomes, but did not exclude variants present solely at low frequency in the ExAC database^15^. In Tables 1 & 2 we included only variants found in all exome-sequenced, affected, family members but not shared by more than one family; this was to exclude variants potentially common to the region not captured in the population resequencing projects. Because samples were not available for all family members, it was not possible to confirm segregation of putatively causal variants for all affected individuals. All variants presented in text were manually visualised to evaluate genotype quality in the raw alignment files using IGV^21^, and no features consistent with errors were present yielding high-confidence genotype calls. The full list of rare (<1% in 1000 Genomes) NS variants classed as damaging by at least one predictive score and potentially damaging splicing variants are given in Supplementary Table 3. Whole-exome genotype calls are provided in Supplementary File 4.

## Results

Table 1 shows likely protein truncating and indel variants in these seven families, with Table 2 listing 28 missense variants. For a given family only variants found for all the exome-sequenced family members (Fig. 1) and classed as deleterious by at least one predictive score is given. Table 2 entries are ordered using combined ranks from most to least deleterious by predictive score^11^. Four of the genes listed in Table 2 (*WNT7A, MSX1, CLPTM1* and *EVC2*, ranked 9, 10, 11 and 23 respectively) have been previously identified as containing variants implicated in NSCLP phenotypes. Family CL1 has the 9^th^ ranked variant in the *WNT7A* gene. Members of the WNT gene family have previously been associated with NSCLP phenotypes^22^,^23^,^24^. Specifically, a number of WNT signalling pathway genes including *WNT3A, WNT5A, WNT9B*, and *WNT11* have been established as candidates^22^ and mouse expression studies have shown roles for WNT genes in mid-facial formation and lip and palate development^25^.

The 10^th^ ranked variant, found in family CL4, is in the *MSX1* gene, and considered damaging by SIFT, PolyPhen-2 and MutationTaster and has high GERP++ and CADD scores. Variants in this gene have been strongly implicated in NSCLP in several studies. Jezewski *et al.*^26^ found mutations in 2% of CLP cases and indicated that this has genetic counselling implications where autosomal dominant inheritance patterns are found. Exon 2 of *MSX1,* in which the p.P260T is located, has been found to be highly conserved with significantly fewer sequence variants compared with exon 1 of this small (two exon) gene^26^. Functional validation of *MSX1* as a candidate is established through a cleft palate and foreshortened maxilla phenotype in knockout mice^27^. A number of association studies have also indicated involvement of *MSX1* in NSCLP^4^,^28^,^29^,^30^,^31^. In a study of 94 patients and 93 controls from Operation Smile, Colombia, four *MSX1* microsatellite alleles were analysed and an increased risk of CLP was observed with CA polymorphisms in the gene^32^. An autosomal dominant *MSX1* mutation in a family with clefting and tooth agenesis showed a familial pattern of segregating *MSX1* mutations^5^. Diverse evidence establishes that *MSX1* promotes growth and inhibits differentiation. Mutations in *MSX1* can cause primary or secondary facial clefting in mouse models^26^.

The 11^th^ ranked variant (from family CL1) is in the *CLPTM1* gene (Cleft lip-and palate-associated transmembrane protein-1) which is situated at 19q13.3. A balanced translocation is this region was found in a multi-case CLP family^33^ and this region is implicated in NSCLP by linkage and transmission disequilibrium test association studies^34^. However a *de novo* deletion of 0.8 Mb in this region associated with CLP, but not encompassing *CLPTM1,* has been reported^35^. As Kohli and Kohli^36^ indicate, the role of *CLPTM1* or other genes in this locus is uncertain.

The 23^rd^ ranked variant is in the *EVC2* gene (family CL2) and belongs to the same two megabase chromosomal region as *MSX1* (4p16). Ingersoll *et al.*^37^ found linkage and association signals in genes in this region. They found suggestive evidence for linkage and association amongst cleft palate trios to *EVC2*. Mutations in *EVC2* can lead to Weyers acrofacial dysostosis^38^, not usually associated with oral clefts but cases with subtle CLP phenotypes, and tooth anomalies have been reported^37^.

## Discussion

Linkage, candidate gene association and genome-wide association (GWAS) have been applied to investigate numerous multifactorial diseases, including NSCLP. As a result of these studies more than 11 genes and gene regions are now known or likely to have an etiologic role in NSCLP^39^. However, there is increasing evidence that NSCLP is a heterogeneous condition comprising a substantial multifactorial component along with a much smaller proportion of cases showing more Mendelian patterns of inheritance. The Gajdos *et al.*^40^ segregation analysis indicated that the complex familial patterns observed in NSCLP is best explained as a mixture of monogenic cases, probably dominantly inherited, combined with others which have a multifactorial aetiology. The conclusions favour analyses of multiple-case pedigrees to reduce heterogeneity and help identify Mendelian sub-forms. Stanier and Moore^41^ identified significant overlaps between genes underlying syndromic and nonsyndromic forms of CLP, recognising that several genes implicated in syndromic disease, including *TBX22, PVRL1, IRF6, P63* and *MSX1*, can also contribute to ~10% of NSCLP. Scapoli *et al.*^42^ point out that the autosomal dominant Van der Woude syndrome (VWS) is only phenotypically distinguished from NSCLP by lower-lip pits and hypodontia which are only variably present in VWS affected individuals. Mutations in the *IRF6* gene, which cause VWS, have been firmly implicated in some NSCLP cases^3^ supporting heterogeneity with the NSCLP clinical designation. Furthermore, Kerameddin *et al.*^43^ found a tag SNP (rs642961) in *IRF6* was associated with the most severe complete bilateral NSCLP phenotype. This suggests multi-case families with bilateral clefts are the most likely to be segregating single gene mutations. This strategy is supported by Vieira *et al.*^44^ who indicate that point mutations in several genes contribute to ~6% of NSCLP, and these are enriched in cases with bilateral clefting.

In Table 2, we identify a coding variant in the *MSX1* gene shared by affected family members in CL4 in which the proband has a bilateral CLP phenotype. Direct sequencing of coding regions has shown rare mutations in *MSX1* may account for ~2% of NSCLP. The identified *MSX1* variant is present at low frequency in the ExAC database (Table 2). ExAC contains >60,000 exomes from various disease specfic and population genetic studies (http://exac.broadinstitute.org/). Functional studies and analyses of larger cohorts of multi-case NSCLP families are required to establish a possible role for this and other rare variants identified in NSCLP phenotypes. Variants identified here also include candidates in the *WNT7A* (family CL1) *,CLPTM1* (family CL1) and *EVC2* genes (family CL2) which should be considered as targets for analysis in additional families.

For investigations aiming to resolve the genetic factors underlying NSCLP in multiple case families, exome sequencing presents a relatively cost-effective approach in which sequencing a small number of affected family members can identify candidate underlying genetic variation. NSCLP provides a particular challenge for genetic studies, with incomplete penetrance and environmental factors hindering the identification of aetiological variance^2^,^39^. We have aimed to minimise this effect by careful selection of pedigrees exhibiting clefting in multiple individuals, where we would expect a stronger genetic component. Filtering power would be increased by the inclusion of further members of the pedigrees, however this has not been viable due to the isolated geographic locations for many individuals.

Exome sequencing yields thousands of variants per individual and identification of candidate variants can only be achieved following extensive filtering. We have undertaken filtering to identify variants predicted as damaging by restricting analysis to a list of 865 genes which have been previously associated with any condition involving CLP. Such an approach risks missing causal variants in novel genes not previously linked to NSCLP, but facilitates practicable data interpretation by virtue of the greater prior probability that they are associated with NSCLP. The composite score based rank using PhyloP, GERP++. CADD and logit (Table 2) has been used successfully prioritise variants involved in syndromic CLP^11^, These four scores are closely correlated, although the composite measures are not independent in every case. Further improvements in predictive tools and recognition of more disease variants and understanding of disease pathways will enable future improvements in interpretation of these complex data sets.

Whilst predictive tools are essential for the prioritisation of variants discovered in next generation sequencing (NGS) studies, ultimately functional validation of the effects of variants on protein function is required to confirm their impact. Given the volume of potentially pathogenic variants being identified in NGS studies, routine functional validation is infeasible. *In silico* protein modelling approaches may also be used to improve throughput, however these require the prior determination of protein structure, which has not been reported in the majority of genes discussed herein. Overall, it is clear that functional validation is a significant bottleneck in NGS studies, and one not readily assuaged.

The limitations of exome sequencing include lack of coverage outside gene coding regions thereby excluding regulatory variants, which may influence risk. Technical limitations include poor coverage of some coding regions thereby missing potential causal variants. Whole genome sequencing offers a solution to these coverage issues, but at higher cost and considerably increased analytical complexity. Given the extent of the missing heritability in CLP, it is likely non-coding regions of the genome play a significant role; whole genome sequencing may therefore provide a valuable tool as sequencing costs continue to drop.

In this study we have limited our analyses to 865 genes with a known/suspected involvement in CLP phenotypes. Whilst this will prevent us from identifying novel aetiological genes, 7 families would be underpowered to identify novel causal genes reliably. Large cohort studies are required in order to identify novel CLP genes; to this end we have made our WES data available in Supplementary File 4 for the use of other researchers.

In conclusion, we have undertaken exome analysis in seven Colombian families with NSCLP phenotypes. We find a deleterious variant in the *MSX1* gene in family CL4 which is a strong candidate for causality. Deleterious variants in at least three additional genes may be implicated in NSCLP phenotypes in some of the other families. Although NSCLP is primarily a complex multifactorial phenotype, our study adds to the growing body of evidence that Mendelian sub-forms exist and these are best studied in multi-case families particularly where there are more severe phenotypic features such as bilateral clefting.

## Additional Information

**How to cite this article**: Pengelly, R. J. *et al.* Deleterious coding variants in multi-case families with non-syndromic cleft lip and/or palate phenotypes. *Sci. Rep.* **6**, 30457; doi: 10.1038/srep30457 (2016).

## Supplementary Material

Supplementary Information

Supplementary Dataset 1

Supplementary Dataset 2

## Figures and Tables

**Table 1:** Protein truncating, splicing and indel variants observed in single families.

Gene	Genomic Position	Transcript ID	Exon	mRNA change	Protein change	Variant type	ExAC MAF	ΔMaxEnt	CL1	CL2	CL3	CL4	CL5	CL6	CL7
*DLG1*	3:196846393	NM_001204388	8	923_925del	308_309del	nonframeshift_deletion	-	.				◊			
*FRAS1*	4:79391228	NM_025074	51	G7354T	E2452X	stopgain	-	.						◊	
*WDR11*	10:122660583	NM_018117	21	2660_2662del	887_888del	nonframeshift_deletion	-	.			◊				
*IGF1R*	15:99500507	NM_000875	21	3940_3941insCGTCCTCCC	L1314delinsPSSL	nonframeshift_insertion	-	.	◊						
*FBLN1*	22:45927140	NM_001996	5	485-5C>-		splicing	-	22	◊						

◊ = Heterozygous variant observed for all family members sequenced.

**Table 2:** Non-synonymous variants observed in single families.

Gene	Genomic Position	Transcript ID	Exon	mRNA change	Protein change	ExAC MAF	SIFT	PolyPhen-2	MutationTaster	Grantham score	PhyloP	GERP++	CADD	Logit	Rank	CL1	CL2	CL3	CL4	CL5	CL6	CL7
*WDR35*	2:20137643	NM_001006657	20	C2161T	R721C	4.1E-05	0.00	0.92	1.00	180	9.81	5.04	27.70	0.13	1	◊						
*PTHLH*	12:28122357	NM_002820	3	G71A	G24E	-	0.00	1.00	0.99	98	5.75	5.13	32.00	0.39	2		◊					
*GPC6*	13:94482686	NM_005708	3	T599A	F200Y	-	0.00	0.98	0.95	22	7.65	5.48	31.00	0.06	3	◊						
*INPPL1*	11:71939494	NM_001567	3	G349A	V117I	-	0.00	0.95	0.04	29	8.18	3.90	22.80	0.11	4	◊						
*MYH3*	17:10539158	NM_002470	29	G3869A	R1290H	3.3E-05	0.00	0.10	0.94	29	4.95	4.84	21.30	0.13	5				◊			
*AHDC1*	1:27876631	NM_001029882	6	C1996G	R666G	8.6E-06	0.00	1.00	0.06	125	8.73	5.08	22.80	0.04	6		◊					
*ABCA12*	2:215928852	NM_173076	3	C254T	T85I	-	0.99	0.73	0.00	89	4.18	5.30	15.26	0.10	7			◊				
*DEAF1*	11:654023	NM_021008	11	C1532G	A511G	-	0.00	0.59	1.00	60	9.01	3.03	17.71	0.08	8			◊				
*WNT7A*	3:13860472	NM_004625	4	G1019A	S340N	-	0.00	0.94	0.99	46	6.07	4.11	23.60	0.06	9	◊						
*MSX1*	4:4864736	NM_002448	2	C778A	P260T	1.3E-04	0.00	0.61	0.99	38	5.96	4.76	27.60	0.04	10				◊			
*CLPTM1*	19:45491357	NM_001294	9	A1058G	N353S	8.2E-06	0.04	0.60	0.99	46	6.60	3.01	17.19	0.09	11	◊						
*IGF1R*	15:99500597	NM_000875	21	C4030G	Q1344E	-	0.00	0.01	0.99	29	4.78	5.24	13.05	0.04	12	◊						
*CFDP1*	16:75429103	NM_006324	5	A535T	T179S	-	0.00	0.02	0.99	58	2.66	5.54	15.68	0.04	13	◊						
*NBAS*	2:15651437	NM_015909	10	G784A	G262S	-	0.01	0.09	0.86	56	4.26	4.15	13.81	0.07	14	◊						
*COL17A1*	10:105795306	NM_000494	49	T3434C	I1145T	1.9E-05	0.00	0.15	0.31	89	5.46	4.39	12.18	0.06	15					◊		
*CDON*	11:125887051	NM_001243597	6	A860G	N287S	-	0.00	0.34	0.64	46	3.10	5.01	15.32	0.04	16							◊
*SNAP29*	22:21224814	NM_004782	2	A427G	N143D	-	0.02	0.34	0.17	23	8.77	3.70	11.41	0.04	17		◊					
*NOTCH2*	1:120509101	NM_001200001	9	G1465T	V489L	-	0.00	0.08	0.34	32	0.87	5.38	12.51	0.05	18				◊			
*MASP1*	3:186937872	NM_001879	16	G2087A	G696E	1.7E-05	0.05	0.09	0.37	98	1.65	3.75	14.53	0.06	19						◊	
*FREM2*	13:39263993	NM_207361	1	A2512G	T838A	8.2E-06	0.00	0.00	1.00	58	2.49	4.44	7.38	0.07	20				◊			
*SPRY4*	5:141693887	NM_030964	3	C856T	R286C	2.5E-05	0.00	0.88	0.97	180	2.44	4.70	13.49	0.04	21			◊				
*ZBTB24*	6:109802863	NM_001164313	2	A367G	K123E	-	0.00	0.05	0.32	56	1.52	4.16	14.67	0.03	22				◊			
*EVC2*	4:5617202	NM_001166136	16	G2536A	E846K	1.6E-05	0.10	0.67	0.27	56	1.14	2.85	16.13	0.03	23		◊					
*SCN2A*	2:166187894	NM_001040143	13	T2204C	M735T	-	0.04	0.00	0.06	81	0.47	2.35	2.95	0.04	24			◊				
*RYR1*	19:38976754	NM_000540	34	G5459T	R1820L	-	0.04	0.01	0.71	102	0.93	1.71	8.87	0.03	25					◊		
*WT1*	11:32456755	NM_024426	1	C137T	A46V	-	0.02	0.00	0.00	64	0.33	0.81	12.21	0.02	26				◊			
*INPPL1*	11:71949096	NM_001567	27	T3563G	L1188R	1.0E-05	0.10	.	0.01	102	0.44	1.47	10.20	0.01	27	◊						
*COL6A2*	21:47551876	NM_001849	28	G2470A	V824M	2.9E-04	0.00	.	1.00	21	.	3.62	.	.	-					◊		

◊ = Heterozygous variant observed for all family members sequenced.

Underlined predictive scores damaging by at least one of: SIFT <0.05 (variant considered to affect protein function); PolyPhen-2 HumVar scores >0.447 (variant possibly damaging) and > = 0.909 (variant probably damaging); MutationTaster scores >0.95 (variant considered damaging); Grantham scores >100 (radical amino acid change).

## References

[b1] Murray J. Gene/environment causes of cleft lip and/or palate. Clin. Genet. 61, 248–256 (2002). 12030886 10.1034/j.1399-0004.2002.610402.x

[b2] Collins A. *et al.* The potential for next-generation sequencing to characterise the genetic variation underlying non-syndromic cleft lip and palate phenotypes. OA Genet. 1, 10 (2013) http://www.oapublishinglondon.com/abstract/987.

[b3] Zucchero T. M. *et al.* Interferon regulatory factor 6 (IRF6) gene variants and the risk of isolated cleft lip or palate. N. Engl. J. Med. 351, 769–780 (2004). 15317890 10.1056/NEJMoa032909

[b4] Lidral A. C. *et al.* Association of MSX1 and TGFB3 with nonsyndromic clefting in humans. Am. J. Hum. Genet. 63, 557–568 (1998). 9683588 10.1086/301956 PMC1377298

[b5] van den Boogaard M. J., Dorland M., Beemer F. A. & van Amstel H. K. MSX1 mutation is associated with orofacial clefting and tooth agenesis in humans. Nat. Genet. 24, 342–343 (2000). 10742093 10.1038/74155

[b6] Leslie E. J. & Marazita M. L. Genetics of cleft lip and cleft palate. Am. J. Med. Genet. C. Semin. Med. Genet. 163C, 246–258 (2013). 24124047 10.1002/ajmg.c.31381 PMC3925974

[b7] Lee S., Wu M. C. & Lin X. Optimal tests for rare variant effects in sequencing association studies. Biostatistics 13, 762–775 (2012). 22699862 10.1093/biostatistics/kxs014 PMC3440237

[b8] Chandrasekharan D. & Ramanathan A. Identification of a novel heterozygous truncation mutation in exon 1 of ARHGAP29 in an Indian subject with nonsyndromic cleft lip with cleft palate. Eur. J. Dent. 8, 528–532 (2014). 25512736 10.4103/1305-7456.143637 PMC4253111

[b9] Blanton S. H. *et al.* Variation in IRF6 contributes to nonsyndromic cleft lip and palate. Am. J. Med. Genet. A 137A, 259–262 (2005). 16096995 10.1002/ajmg.a.30887

[b10] Pengelly R. J. *et al.* A SNP profiling panel for sample tracking in whole-exome sequencing studies. Genome Med. 5, 89 (2013). 24070238 10.1186/gm492 PMC3978886

[b11] Pengelly R. J. *et al.* Resolving clinical diagnoses for syndromic cleft lip and/or palate phenotypes using whole-exome sequencing. Clin Genet 88, 441–449 (2015). 25441681 10.1111/cge.12547

[b12] Stenson P. D. *et al.* The Human Gene Mutation Database: building a comprehensive mutation repository for clinical and molecular genetics, diagnostic testing and personalized genomic medicine. Hum. Genet. 133, 1–9 (2014). 24077912 10.1007/s00439-013-1358-4 PMC3898141

[b13] Hamosh A., Scott A. F., Amberger J. S., Bocchini C. A. & McKusick V. A. Online Mendelian Inheritance in Man (OMIM), a knowledgebase of human genes and genetic disorders. Nucleic Acids Res. 33, D514–D517 (2005). 15608251 10.1093/nar/gki033 PMC539987

[b14] Liu X., Jian X. & Boerwinkle E. dbNSFP: a lightweight database of human nonsynonymous SNPs and their functional predictions. Hum. Mutat. 32, 894–899 (2011). 21520341 10.1002/humu.21517 PMC3145015

[b15] Exome Aggregation Consortium *et al.* Analysis of protein-coding genetic variation in 60,706 humans. bioRxiv 030338 doi: 10.1101/030338 (2015)

[b16] Yeo G. & Burge C. B. Maximum entropy modeling of short sequence motifs with applications to RNA splicing signals. J. Comput. Biol. 11, 377–394 (2004). 15285897 10.1089/1066527041410418

[b17] Fuentes Fajardo K. V. *et al.* Detecting false-positive signals in exome sequencing. Hum. Mutat. 33, 609–613 (2012). 22294350 10.1002/humu.22033 PMC3302978

[b18] Sherry S. T. *et al.* dbSNP: the NCBI database of genetic variation. Nucleic Acids Res. 29, 308–311 (2001). 11125122 10.1093/nar/29.1.308 PMC29783

[b19] Abecasis G. R. *et al.* An integrated map of genetic variation from 1,092 human genomes. Nature 491, 56–65 (2012). 23128226 10.1038/nature11632 PMC3498066

[b20] Fu W. *et al.* Analysis of 6,515 exomes reveals the recent origin of most human protein-coding variants. Nature 493, 216–220 (2013). 23201682 10.1038/nature11690 PMC3676746

[b21] Thorvaldsdóttir H., Robinson J. T. & Mesirov J. P. Integrative Genomics Viewer (IGV): high-performance genomics data visualization and exploration. Brief. Bioinform. 14, 178–192 (2013). 22517427 10.1093/bib/bbs017 PMC3603213

[b22] Chiquet B. T. *et al.* Variation in WNT genes is associated with non-syndromic cleft lip with or without cleft palate. Hum. Mol. Genet. 17, 2212–2218 (2008). 18413325 10.1093/hmg/ddn121 PMC2852032

[b23] Menezes R. *et al.* Studies with Wnt genes and nonsyndromic cleft lip and palate. Birth Defects Res. A. Clin. Mol. Teratol. 88, 995–1000 (2010). 20890934 10.1002/bdra.20720 PMC2991560

[b24] Mostowska A. *et al.* Genotype and haplotype analysis of WNT genes in non-syndromic cleft lip with or without cleft palate. Eur. J. Oral Sci. 120, 1–8 (2012). 22288914 10.1111/j.1600-0722.2011.00938.x

[b25] Lan Y. *et al.* Expression of Wnt9b and activation of canonical Wnt signaling during midfacial morphogenesis in mice. Dev. Dyn. 235, 1448–1454 (2006). 16496313 10.1002/dvdy.20723 PMC2559872

[b26] Jezewski P. A. *et al.* Complete sequencing shows a role for MSX1 in non-syndromic cleft lip and palate. J. Med. Genet. 40, 399–407 (2003). 12807959 10.1136/jmg.40.6.399 PMC1735501

[b27] Satokata I. & Maas R. Msx1 deficient mice exhibit cleft palate and abnormalities of craniofacial and tooth development. Nat. Genet. 6, 348–356 (1994). 7914451 10.1038/ng0494-348

[b28] Romitti P. A. *et al.* Candidate genes for nonsyndromic cleft lip and palate and maternal cigarette smoking and alcohol consumption: Evaluation of genotype-environment interactions from a population-based case-control study of orofacial clefts. Teratology 59, 39–50 (1999). 9988882 10.1002/(SICI)1096-9926(199901)59:1<39::AID-TERA9>3.0.CO;2-7

[b29] Blanco R. *et al.* Evidence of a sex-dependent association between the MSX1 locus and nonsyndromic cleft lip with or without cleft palate in the Chilean population. Hum. Biol. 73, 81–89 (2001). 11332647 10.1353/hub.2001.0002

[b30] Beaty T. H. *et al.* A Case-Control Study of Nonsyndromic Oral Clefts in Maryland. Ann. Epidemiol. 11, 434–442 (2001). 11454503 10.1016/s1047-2797(01)00222-8

[b31] Jugessur A. *et al.* Variants of developmental genes (TGFA, TGFB3, and MSX1) and their associations with orofacial clefts: a case-parent triad analysis. Genet. Epidemiol. 24, 230–239 (2003). 12652527 10.1002/gepi.10223

[b32] Otero L., Gutiérrez S., Cháves M., Vargas C. & Bérmudez L. Association of MSX1 with nonsyndromic cleft lip and palate in a Colombian population. Cleft Palate. Craniofac. J. 44, 653–656 (2007). 18177186 10.1597/06-097.1

[b33] Yoshiura K. *et al.* Characterization of a novel gene disrupted by a balanced chromosomal translocation t(2;19)(q11.2;q13.3) in a family with cleft lip and palate. Genomics 54, 231–240 (1998). 9828125 10.1006/geno.1998.5577

[b34] Wyszynski D. F. *et al.* Evidence for an association between markers on chromosome 19q and non-syndromic cleft lip with or without cleft palate in two groups of multiplex families. Hum. Genet. 99, 22–26 (1997). 9003487 10.1007/s004390050303

[b35] Leal T. *et al.* Array-CGH detection of a de novo 0.8 Mb deletion in 19q13.32 associated with mental retardation, cardiac malformation, cleft lip and palate, hearing loss and multiple dysmorphic features. Eur. J. Med. Genet. 52, 62–66 (2009). 19022414 10.1016/j.ejmg.2008.09.007

[b36] Kohli S. S. & Kohli V. S. A comprehensive review of the genetic basis of cleft lip and palate. J. Oral Maxillofac. Pathol. 16, 64–72 (2012). 22438645 10.4103/0973-029X.92976 PMC3303526

[b37] Ingersoll R. G. *et al.* Association between genes on chromosome 4p16 and non-syndromic oral clefts in four populations. Eur. J. Hum. Genet. 18, 726–732 (2010). 20087401 10.1038/ejhg.2009.228 PMC2874614

[b38] D’Asdia M. C. *et al.* Novel and recurrent EVC and EVC2 mutations in Ellis-van Creveld syndrome and Weyers acrofacial dyostosis. Eur. J. Med. Genet. 56, 80–87 (2013). 23220543 10.1016/j.ejmg.2012.11.005

[b39] Dixon M. J., Marazita M. L., Beaty T. H. & Murray J. C. Cleft lip and palate: understanding genetic and environmental influences. Nat. Rev. Genet. 12, 167–178 (2011). 21331089 10.1038/nrg2933 PMC3086810

[b40] Gajdos V. *et al.* Genetics of nonsyndromic cleft lip with or without cleft palate: is there a Mendelian sub-entity? Ann. génétique 47, 29–39 (2004). 10.1016/j.anngen.2003.12.002 15050872

[b41] Stanier P. & Moore G. E. Genetics of cleft lip and palate: syndromic genes contribute to the incidence of non-syndromic clefts. Hum. Mol. Genet. 13, R73–R81 (2004). 14722155 10.1093/hmg/ddh052

[b42] Scapoli L. *et al.* Strong evidence of linkage disequilibrium between polymorphisms at the IRF6 locus and nonsyndromic cleft lip with or without cleft palate, in an Italian population. Am. J. Hum. Genet. 76, 180–183 (2005). 15558496 10.1086/427344 PMC1196422

[b43] Kerameddin S., Namipashaki A., Ebrahimi S. & Ansari-Pour N. IRF6 Is a Marker of Severity in Nonsyndromic Cleft Lip/Palate. J. Dent. Res. 94, 226S–232S (2015). 25896061 10.1177/0022034515581013

[b44] Vieira A. R. *et al.* Medical sequencing of candidate genes for nonsyndromic cleft lip and palate. PLoS Genet. 1, e64 (2005). 16327884 10.1371/journal.pgen.0010064 PMC1298935

