# Supplementary material for: Deleterious coding variants in multi-case families with non-syndromic cleft lip and/or palate phenotypes
Source: Sci Rep. 2016 Jul 26;6:30457. doi: 10.1038/srep30457 (PMC4960602; doi:10.1038/srep30457)
Supplement: Supplementary Information [file srep30457-s1.pdf]

Deleterious coding variants in multi-case families with non-syndromic cleft lip and/or palate phenotypes.

Reuben J. Pengelly, Liliana Arias, Julio Martínez, Rosanna Upstill-Goddard, Eleanor G. Seaby, Jane Gibson, Sarah Ennis, Andrew Collins & Ignacio Briceño

Table S1 | Depth of coverage statistics for exome sequence data

Sample	CL1_1	CL1_2	CL2_1	CL2_2	CL3_1	CL4_1	CL4_2	CL5_1	CL6_1	CL6_2	CL7_1	CL7_2
Total no. sequence reads	54,956,672	74,355,880	53,630,678	46,509,220	43,363,414	41,622,776	46,087,918	49,254,976	100,068,158	99,974,938	95,428,304	97,299,918
Total no. aligned reads	54,558,769	73,326,222	53,245,840	46,179,091	43,032,505	41,312,749	45,762,033	48,875,805	99,055,310	98,831,948	94,564,501	96,408,611
Total no. unique alignments	53,763,584	72,878,819	52,373,414	45,401,788	42,300,126	40,618,226	44,987,196	48,031,749	97,692,151	97,384,564	93,168,229	95,022,993
Mapped to target reads +/- 150bp (%)	91.13	95.19	88.21	87.93	87.89	88.2	87.56	84.46	91.54	87.17	88.3	89.82
Mapped to target reads (%)	76.81	84.36	74.84	75.31	75.34	75.5	74.41	71.99	86.61	81.75	84.74	85.7
Target bases with coverage >1 (%)	99.34	99.87	99.32	99.2	99.18	99.16	99.18	99.31	99.35	99.38	99.25	99.27
Target bases with coverage >5 (%)	98.84	99.33	98.69	98.41	98.27	98.2	98.39	98.48	99.06	99.13	98.96	98.99
Target bases with coverage >10 (%)	97.74	98.13	97.14	96.42	95.72	95.48	96.28	96.36	98.62	98.75	98.54	98.6
Target bases with coverage >20 (%)	92.92	93.67	90.68	88.26	85.87	85.04	87.83	88.18	96.93	97.38	97.14	97.34
Mean read depth across exome	68.84	94.55	65.22	57.33	52.62	50.94	56.61	59.21	109.24	119.05	118.47	123.11

Table S2 | 865 genes associated with cleft lip/palate phenotypes

Gene Symbol	Gene name	Locus
ABCA12	ATP-binding cassette, sub-family A (ABC1), member 12	2q34
ABCA3	ATP-binding cassette, sub-family A (ABC1), member 3	16p13.3
ABCA4	ATP-binding cassette, sub-family A (ABC1), member 4	1p22
ABCB1	ATP-binding cassette, sub-family B (MDR/TAP), member 1	7q21.12
ABCC6	ATP-binding cassette, sub-family C (CFTR/MRP), member 6	16p13.11
ABCD4	ATP-binding cassette, sub-family D (ALD), member 4	14q24
ABO	ABO blood group (transferase A, alpha 1-3-N-acetylgalactosaminyltransferase; transferase B, alpha 1-3-galactosyltransferase)	9q34.2
ACAN	aggrecan	15q26.1
ACHE	acetylcholinesterase (Yt blood group)	7q22
ACOX1	acyl-CoA oxidase 1, palmitoyl	17q25.1
ACTA1	actin, alpha 1, skeletal muscle	1q42.13
ACTB	actin, beta	7p22
ADAMTS10	ADAM metallopeptidase with thrombospondin type 1 motif, 10	19p13.2
ADAMTS17	ADAM metallopeptidase with thrombospondin type 1 motif, 17	15q24
ADAMTS2	ADAM metallopeptidase with thrombospondin type 1 motif, 2	5q23-q24
ADAMTSL2	ADAMTS-like 2	9q34.3
ADH1A	alcohol dehydrogenase 1A (class I), alpha polypeptide	4q23
ADNP	activity-dependent neuroprotector homeobox	20q13.13
AFF2	AF4/FMR2 family, member 2	Xq28
AGPS	alkylglycerone phosphate synthase	2q
AHDC1	AT hook, DNA binding motif, containing 1	1p36.13
AIC	Aicardi syndrome	Xp22
AKT1	v-akt murine thymoma viral oncogene homolog 1	14q32.33
AKT3	v-akt murine thymoma viral oncogene homolog 3	1q44
ALDH18A1	aldehyde dehydrogenase 18 family, member A1	10q24.3-q24.6
ALG1	ALG1, chitobiosyldiphosphodolichol beta-mannosyltransferase	16p13.3
ALG12	ALG12, alpha-1,6-mannosyltransferase	22q13.33

ALG6	ALG6, alpha-1,3-glucosyltransferase	1p31.3
ALG9	ALG9, alpha-1,2-mannosyltransferase	11q23
ALX1	ALX homeobox 1	12q21.31
ALX3	ALX homeobox 3	1p13.3
ALX4	ALX homeobox 4	11p11.2
AMER1	APC membrane recruitment protein 1	Xq11.1
ANK1	ankyrin 1, erythrocytic	8p11.21
ANKH	ANKH inorganic pyrophosphate transport regulator	5p15.2
ANKRD11	ankyrin repeat domain 11	16q24.3
ANTXR1	anthrax toxin receptor 1	2p13.1
ANTXR2	anthrax toxin receptor 2	4q21.3
APC	adenomatous polyposis coli	5q21-q22
ARHGAP29	Rho GTPase activating protein 29	1p22.1
ARHGAP31	Rho GTPase activating protein 31	3q13.33
ARHGEF9	Cdc42 guanine nucleotide exchange factor (GEF) 9	Xq11.1
ARID1B	AT rich interactive domain 1B (SWI1-like)	6q25.3
ARL6	ADP-ribosylation factor-like 6	3q11.2
ARNT	aryl hydrocarbon receptor nuclear translocator	1q21
ARX	aristaless related homeobox	Xp21.3
ASNS	asparagine synthetase (glutamine-hydrolyzing)	7q21.3
ASPH	aspartate beta-hydroxylase	8q12.1
ASXL1	additional sex combs like transcriptional regulator 1	20q11
ASXL3	additional sex combs like transcriptional regulator 3	18q11
ATD	asphixiating thoracic dystrophy (chondroectodermal dysplasia-like syndrome)	12p12.2-p11.21
ATIC	5-aminoimidazole-4-carboxamide ribonucleotide formyltransferase/IMP cyclohydrolase	2q35
ATL1	atlastin GTPase 1	14q21.3
ATP6V0A2	ATPase, H+ transporting, lysosomal V0 subunit a2	12q24.31
ATP7A	ATPase, Cu++ transporting, alpha polypeptide	Xq21.1
ATPAF2	ATP synthase mitochondrial F1 complex assembly factor 2	17p11.2
ATR	ATR serine/threonine kinase	3q23

ATRNL1	attractin-like 1	10q26
ATRX	alpha thalassemia/mental retardation syndrome X-linked	Xq21.1
AUTS2	autism susceptibility candidate 2	7q11.22
B3GALT6	UDP-Gal:betaGal beta 1,3-galactosyltransferase polypeptide 6	1p36.33
B3GALTL	beta 1,3-galactosyltransferase-like	13q12.3
B3GAT3	beta-1,3-glucuronyltransferase 3	11q12
B4GALT7	xylosylprotein beta 1,4-galactosyltransferase, polypeptide 7	5q35.1-q35.3
B9D1	B9 protein domain 1	17p11.2
BANF1	barrier to autointegration factor 1	11q13.1
BBIP1	BBSome interacting protein 1	10q25.3
BBS1	Bardet-Biedl syndrome 1	11q13
BBS10	Bardet-Biedl syndrome 10	12q21.2
BBS12	Bardet-Biedl syndrome 12	4q27
BBS2	Bardet-Biedl syndrome 2	16q21
BBS4	Bardet-Biedl syndrome 4	15q22.3-q23
BBS5	Bardet-Biedl syndrome 5	2q31
BBS7	Bardet-Biedl syndrome 7	4q27
BBS9	Bardet-Biedl syndrome 9	7p14
BCOR	BCL6 corepressor	Xp11.4
BCS1L	BC1 (ubiquinol-cytochrome c reductase) synthesis-like	2q35
BEST1	bestrophin 1	11q12
BLM	Bloom syndrome, RecQ helicase-like	15q26.1
BMP1	bone morphogenetic protein 1	8p21
BMP2	bone morphogenetic protein 2	20p12
BMP4	bone morphogenetic protein 4	14q22-q23
BMPER	BMP binding endothelial regulator	7p14.3
BMPR1A	bone morphogenetic protein receptor, type IA	10q22.3
BMPR1B	bone morphogenetic protein receptor, type IB	4q23-q24
BRAF	B-Raf proto-oncogene, serine/threonine kinase	7q34
BRIP1	BRCA1 interacting protein C-terminal helicase 1	17q22.2

BUB1B	BUB1 mitotic checkpoint serine/threonine kinase B	15q15
C12orf57	chromosome 12 open reading frame 57	12p13.31
C15orf41	chromosome 15 open reading frame 41	15q14
C5orf42	chromosome 5 open reading frame 42	5p13.2
C6	complement component 6	5p13.1
CACNA1C	calcium channel, voltage-dependent, L type, alpha 1C subunit	12p13.3
CAMK2G	calcium/calmodulin-dependent protein kinase II gamma	10q22
CANT1	calcium activated nucleotidase 1	17q25.3
CAPN5	calpain 5	11q14
CASK	calcium/calmodulin-dependent serine protein kinase (MAGUK family)	Xp11.4
CASP7	caspase 7, apoptosis-related cysteine peptidase	10q25
CAV3	caveolin 3	3p25
CBFB	core-binding factor, beta subunit	16q22.1
CC2D2A	coiled-coil and C2 domain containing 2A	4p15.33
CCBE1	collagen and calcium binding EGF domains 1	18q21.32
CCM2	cerebral cavernous malformation 2	7p13
CD96	CD96 molecule	3p13-q13.2
CDAN1	codanin 1	15q15.2
CDC6	cell division cycle 6	17q21.3
CDH1	cadherin 1, type 1, E-cadherin (epithelial)	16q22.1
CDH15	cadherin 15, type 1, M-cadherin (myotubule)	16q24.3
CDH3	cadherin 3, type 1, P-cadherin (placental)	16q22.1
CDH8	cadherin 8, type 2	16q22.1
CDKN1C	cyclin-dependent kinase inhibitor 1C (p57, Kip2)	11p15.5
CDON	cell adhesion associated, oncogene regulated	11q24.2
CEP290	centrosomal protein 290kDa	12q21.33
CEP57	centrosomal protein 57kDa	11q21
CEP89	centrosomal protein 89kDa	19q13.11
CERS3	ceramide synthase 3	15q26.3
CFDP1	craniofacial development protein 1	16q22.2-q22.3

CFTR	cystic fibrosis transmembrane conductance regulator (ATP-binding cassette sub-family C, member 7)	7q31-q32
CHD2	chromodomain helicase DNA binding protein 2	15q26
CHD6	chromodomain helicase DNA binding protein 6	20q12
CHD7	chromodomain helicase DNA binding protein 7	8q12.2
CHM	choroideremia (Rab escort protein 1)	Xq21.1-q21.3
CHRNA1	cholinergic receptor, nicotinic, alpha 1 (muscle)	2q31.1
CHRNA7	cholinergic receptor, nicotinic, alpha 7 (neuronal)	15q13.3
CHRND	cholinergic receptor, nicotinic, delta (muscle)	2q37.1
CHRNE	cholinergic receptor, nicotinic, epsilon (muscle)	17p13.2
CHRNG	cholinergic receptor, nicotinic, gamma (muscle)	2q37.1
CHST14	carbohydrate (N-acetylgalactosamine 4-0) sulfotransferase 14	15q15.1
CHST3	carbohydrate (chondroitin 6) sulfotransferase 3	10q22.1
CHSY1	chondroitin sulfate synthase 1	15q26.3
CHUK	conserved helix-loop-helix ubiquitous kinase	10q24-q25
CLAM	cerebellar atrophy with progressive microcephaly	7q11-q21
CLCF1	cardiotrophin-like cytokine factor 1	11q13.3
CLPTM1	cleft lip and palate associated transmembrane protein 1	19q13.3
CLPTM1L	CLPTM1-like	5p15.33
CNTNAP2	contactin associated protein-like 2	7q35
COG1	component of oligomeric golgi complex 1	17q25.1
COG7	component of oligomeric golgi complex 7	16p12.2
COL11A1	collagen, type XI, alpha 1	1p21
COL11A2	collagen, type XI, alpha 2	6p21.3
COL17A1	collagen, type XVII, alpha 1	10q24.3
COL1A1	collagen, type I, alpha 1	17q21.33
COL1A2	collagen, type I, alpha 2	7q21.3
COL26A1	collagen, type XXVI, alpha 1	7q22.1
COL2A1	collagen, type II, alpha 1	12q12-q13.2
COL6A2	collagen, type VI, alpha 2	21q22.3
COL7A1	collagen, type VII, alpha 1	3p21.1

COLEC11	collectin sub-family member 11	2p25.3
COLQ	collagen-like tail subunit (single strand of homotrimer) of asymmetric acetylcholinesterase	3p
COMP	cartilage oligomeric matrix protein	19p13.1
COMT	catechol-O-methyltransferase	22q11.21
COQ2	coenzyme Q2 4-hydroxybenzoate polyprenyltransferase	4q21.23
CPT2	carnitine palmitoyltransferase 2	1p32.3
CREBBP	CREB binding protein	16p13.3
CRIPT	cysteine-rich PDZ-binding protein	2p21
CRISPLD2	cysteine-rich secretory protein LCCL domain containing 2	16q24.1
CRLF1	cytokine receptor-like factor 1	19p12
CTAG1A	cancer/testis antigen 1A	Xq28
CTAG1B	cancer/testis antigen 1B	Xq28
CTAG2	cancer/testis antigen 2	Xq28
CTCF	CCCTC-binding factor (zinc finger protein)	16q21-q22.3
CTDP1	CTD (carboxy-terminal domain, RNA polymerase II, polypeptide A) phosphatase, subunit 1	18q23
CUL4B	cullin 4B	Xq23
CUL7	cullin 7	6p21.1
CYP11A1	cytochrome P450, family 11, subfamily A, polypeptide 1	15q23-q24
CYP17A1	cytochrome P450, family 17, subfamily A, polypeptide 1	10q24.3
CYP19A1	cytochrome P450, family 19, subfamily A, polypeptide 1	15q21
CYP1B1	cytochrome P450, family 1, subfamily B, polypeptide 1	2p22.2
CYP26B1	cytochrome P450, family 26, subfamily B, polypeptide 1	2p12
CYP26C1	cytochrome P450, family 26, subfamily C, polypeptide 1	10q23.33
D2HGDH	D-2-hydroxyglutarate dehydrogenase	2p25.3
DAB1	Dab, reelin signal transducer, homolog 1 (Drosophila)	1p32-p31
DCHS1	dachsous cadherin-related 1	11p15.4
DDR1	discoidin domain receptor tyrosine kinase 1	6p21.33
DDR2	discoidin domain receptor tyrosine kinase 2	1q12-q23
DDX11	DEAD/H (Asp-Glu-Ala-Asp/His) box helicase 11	12p11.21
DDX59	DEAD (Asp-Glu-Ala-Asp) box polypeptide 59	1q32.1

DEAF1	DEAF1 transcription factor	11p15.5
DHCR24	24-dehydrocholesterol reductase	1p32.3
DHCR7	7-dehydrocholesterol reductase	11q13.4
DHODH	dihydroorotate dehydrogenase (quinone)	16q22.2
DIH1	diaphragmatic hernia 1	15q26.1-q26.2
DIS3L2	DIS3 like 3'-5' exoribonuclease 2	2q37.1
DISC1	disrupted in schizophrenia 1	1q42.1
DISP1	dispatched homolog 1 (Drosophila)	1q42.12
DKC1	dyskeratosis congenita 1, dyskerin	Xq28
DLG1	discs, large homolog 1 (Drosophila)	3q29
DLG2	discs, large homolog 2 (Drosophila)	11q21
DLX2	distal-less homeobox 2	2q31.1
DLX3	distal-less homeobox 3	17q21.33
DLX5	distal-less homeobox 5	7q21.3
DLX6	distal-less homeobox 6	7q21.3
DNM2	dynamin 2	19p
DNMT3B	DNA (cytosine-5-)-methyltransferase 3 beta	20q11.2
DOCK6	dedicator of cytokinesis 6	19p13.2
DOK7	docking protein 7	4p16.2
DPAGT1	dolichyl-phosphate (UDP-N-acetylglucosamine) N-acetylglucosaminephosphotransferase 1 (GlcNAc-1-P transferase)	11q23.3
DPM1	dolichyl-phosphate mannosyltransferase polypeptide 1, catalytic subunit	20q13.1
DPM2	dolichyl-phosphate mannosyltransferase polypeptide 2, regulatory subunit	9q34.13
DPYD	dihydropyrimidine dehydrogenase	1p22
DPYS	dihydropyrimidinase	8q22
DURS1	Duane retraction syndrome 1	8q13
DUSP22	dual specificity phosphatase 22	6p25.3
DUSP6	dual specificity phosphatase 6	12q22-q23
DYNC1H1	dynein, cytoplasmic 1, heavy chain 1	14q32.31
DYNC2H1	dynein, cytoplasmic 2, heavy chain 1	11q21-q22.1
DYRK1A	dual-specificity tyrosine-(Y)-phosphorylation regulated kinase 1A	21q22.13

EARS2	glutamyl-tRNA synthetase 2, mitochondrial	16p12.2
EBM	epidermolysis bullosa, macular type	X
EBP	emopamil binding protein (sterol isomerase)	Xp11.23-p11.22
ECE1	endothelin converting enzyme 1	1p36.1
ECEL1	endothelin converting enzyme-like 1	2q37.1
EDA2R	ectodysplasin A2 receptor	Xq11.1
EDAR	ectodysplasin A receptor	2q13
EDN1	endothelin 1	6p24.1
EEC1	ectrodactyly, ectodermal dysplasia and cleft lip/palate syndrome 1	7q11.2-q21.3
EFEMP2	EGF containing fibulin-like extracellular matrix protein 2	11q13
EFNB1	ephrin-B1	Xq12
EFTUD2	elongation factor Tu GTP binding domain containing 2	17q21.31
EIF4A3	eukaryotic translation initiation factor 4A3	17q25.3
ELP4	elongator acetyltransferase complex subunit 4	11p13
EMG1	EMG1 N1-specific pseudouridine methyltransferase	12p13
EMX2	empty spiracles homeobox 2	10q26.11
ENG	endoglin	9q34.11
EP300	E1A binding protein p300	22q13.2
EPCAM	epithelial cell adhesion molecule	2p21
EPG5	ectopic P-granules autophagy protein 5 homolog (C. elegans)	18q12.3
EPHA3	EPH receptor A3	3p11.2
EPHA7	EPH receptor A7	6q16.3
EPHX1	epoxide hydrolase 1, microsomal (xenobiotic)	1q42.1
ERBB3	v-erb-b2 avian erythroblastic leukemia viral oncogene homolog 3	12q13
ERCC1	excision repair cross-complementation group 1	19q13.32
ERCC2	excision repair cross-complementation group 2	19q13.3
ERCC5	excision repair cross-complementation group 5	13q22-q34
ERCC6	excision repair cross-complementation group 6	10q11
ERF	Ets2 repressor factor	19q13
ESCO2	establishment of sister chromatid cohesion N-acetyltransferase 2	8p21.1

ESR1	estrogen receptor 1	6q24-q27
EVC	Ellis van Creveld syndrome	4p16
EVC2	Ellis van Creveld syndrome 2	4p16.2-p16.1
EXPH5	exophilin 5	11q22.3
EYA1	EYA transcriptional coactivator and phosphatase 1	8q13.3
EZH2	enhancer of zeste 2 polycomb repressive complex 2 subunit	7q35-q36
F13A1	coagulation factor XIII, A1 polypeptide	6p24.2-p23
F8	coagulation factor VIII, procoagulant component	Xq28
FAF1	Fas (TNFRSF6) associated factor 1	1p32.3
FAM111A	family with sequence similarity 111, member A	11q12.1
FAM111B	family with sequence similarity 111, member B	11q12.1
FAM20C	family with sequence similarity 20, member C	7p22.3
FAM58A	family with sequence similarity 58, member A	Xq28
FANCA	Fanconi anemia, complementation group A	16q24.3
FANCE	Fanconi anemia, complementation group E	6p22-p21
FAT4	FAT atypical cadherin 4	4q28.1
FBLN1	fibulin 1	22q13.31
FBN1	fibrillin 1	15q21.1
FBXL4	F-box and leucine-rich repeat protein 4	6q16.1-q16.3
FERMT1	fermitin family member 1	20p12.3
FGD1	FYVE, RhoGEF and PH domain containing 1	Xp11.21
FGF10	fibroblast growth factor 10	5p13-p12
FGF16	fibroblast growth factor 16	Xq21.1
FGF17	fibroblast growth factor 17	8p21.3
FGF8	fibroblast growth factor 8 (androgen-induced)	10q25-q26
FGFR1	fibroblast growth factor receptor 1	8p11.23-p11.22
FGFR2	fibroblast growth factor receptor 2	10q25.3-q26
FGFR3	fibroblast growth factor receptor 3	4p16.3
FGFRL1	fibroblast growth factor receptor-like 1	4p16
FIG4	FIG4 phosphoinositide 5-phosphatase	6q21

FLNA	filamin A, alpha	Xq28
FLNB	filamin B, beta	3p14.3
FLRT3	fibronectin leucine rich transmembrane protein 3	20p11
FLVCR2	feline leukemia virus subgroup C cellular receptor family, member 2	14q24.3
FMN1	formin 1	15q13.3
FMR1	fragile X mental retardation 1	Xq27.3
FOXC1	forkhead box C1	6p25
FOXC2	forkhead box C2 (MFH-1, mesenchyme forkhead 1)	16q24.1
FOXE1	forkhead box E1 (thyroid transcription factor 2)	9q22
FOXF1	forkhead box F1	16q24
FOXF2	forkhead box F2	6p25.3
FOXL2	forkhead box L2	3q23
FOXP2	forkhead box P2	7q31
FRAS1	Fraser extracellular matrix complex subunit 1	4q21.21
FREM1	FRAS1 related extracellular matrix 1	9p22.3
FREM2	FRAS1 related extracellular matrix protein 2	13q13.3
FTO	fat mass and obesity associated	16q12.2
G6PC3	glucose 6 phosphatase, catalytic, 3	17q21.31
GAA	glucosidase, alpha; acid	17q25.2-q25.3
GABRB3	gamma-aminobutyric acid (GABA) A receptor, beta 3	15q12
GAD1	glutamate decarboxylase 1 (brain, 67kDa)	2q31
GAS1	growth arrest-specific 1	9q21.3-q22
GATA2	GATA binding protein 2	3q21
GATA3	GATA binding protein 3	10p15
GBA	glucosidase, beta, acid	1q22
GCK	glucokinase (hexokinase 4)	7p15.3-p15.1
GCLC	glutamate-cysteine ligase, catalytic subunit	6p12
GDF5	growth differentiation factor 5	20q11.2
GDF6	growth differentiation factor 6	8q22.1
GGH	gamma-glutamyl hydrolase (conjugase, folylpolygammaglutamyl hydrolase)	8q12.3

GH1	growth hormone 1	17q22-q24
GHRHR	growth hormone releasing hormone receptor	7p14
GJA1	gap junction protein, alpha 1, 43kDa	6q22.31
GJB2	gap junction protein, beta 2, 26kDa	13q11-q12
GJB3	gap junction protein, beta 3, 31kDa	1p34
GJB4	gap junction protein, beta 4, 30.3kDa	1p35-p34
GJB6	gap junction protein, beta 6, 30kDa	13q12
GK	glycerol kinase	Xp21.3
GLE1	GLE1 RNA export mediator	9q34.13
GLI2	GLI family zinc finger 2	2q14
GLI3	GLI family zinc finger 3	7p13
GMPPB	GDP-mannose pyrophosphorylase B	3p21.31
GNAI3	guanine nucleotide binding protein (G protein), alpha inhibiting activity polypeptide 3	1p13
GNAS	GNAS complex locus	20q13.2-q13.3
GNAS-AS1	GNAS antisense RNA 1	20q13.32
GNAT2	guanine nucleotide binding protein (G protein), alpha transducing activity polypeptide 2	1p13
GNPAT	glyceronephosphate O-acyltransferase	1q42
GNPTAB	N-acetylglucosamine-1-phosphate transferase, alpha and beta subunits	12q23.3
GNRH1	gonadotropin-releasing hormone 1 (luteinizing-releasing hormone)	8p21-p11.2
GNRHR	gonadotropin-releasing hormone receptor	4q21.2
GOSR2	golgi SNAP receptor complex member 2	17q21
GPC3	glypican 3	Xq26
GPC6	glypican 6	13q32
GPR143	G protein-coupled receptor 143	Xp22.3
GPSM2	G-protein signaling modulator 2	1p13.3
GRB10	growth factor receptor-bound protein 10	7p12.2
GREM1	gremlin 1, DAN family BMP antagonist	15q13.3
GRHL3	grainyhead-like 3 (Drosophila)	1p36
GRIA3	glutamate receptor, ionotropic, AMPA 3	Xq25
GRIN1	glutamate receptor, ionotropic, N-methyl D-aspartate 1	9q34.3

GRIN2A	glutamate receptor, ionotropic, N-methyl D-aspartate 2A	16p13.2
GRIP1	glutamate receptor interacting protein 1	12q13.13
GSC	goosecoid homeobox	14q32.13
GSK3B	glycogen synthase kinase 3 beta	3q13.3
GTF2IRD1	GTF2I repeat domain containing 1	7q11.23
GUSB	glucuronidase, beta	7q11.21
H19	H19, imprinted maternally expressed transcript (non-protein coding)	11p15.5
HAMP	hepcidin antimicrobial peptide	19q13.1
HCN4	hyperpolarization activated cyclic nucleotide-gated potassium channel 4	15q24.1
HDAC4	histone deacetylase 4	2q37.3
HDAC6	histone deacetylase 6	Xp11.23
HDAC8	histone deacetylase 8	Xq13
HESX1	HESX homeobox 1	3p14.3
HFE	hemochromatosis	6p21.3
HIC1	hypermethylated in cancer 1	17p13.3
HMBS	hydroxymethylbilane synthase	11q23.3
HMX1	H6 family homeobox 1	4p16.1
HOXA13	homeobox A13	7p15.2
HOXA2	homeobox A2	7p15.2
HOXB1	homeobox B1	17q21.32
HOXB6	homeobox B6	17q21.32
HOXD1	homeobox D1	2q31.1
HOXD13	homeobox D13	2q31.1
HPE1	holoprosencephaly 1, alobar	21q22.3
HPGD	hydroxyprostaglandin dehydrogenase 15-(NAD)	4q34-q35
HRAS	Harvey rat sarcoma viral oncogene homolog	11p15.5
HS6ST1	heparan sulfate 6-O-sulfotransferase 1	2q21
HSD17B4	hydroxysteroid (17-beta) dehydrogenase 4	5q2
HSPG2	heparan sulfate proteoglycan 2	1p36.1-p35
HYAL1	hyaluronoglucosaminidase 1	3p21.31

HYLS1	hydrolethalus syndrome 1	11q24
ICK	intestinal cell (MAK-like) kinase	6p12.3-p11.2
IDS	iduronate 2-sulfatase	Xq27.3-q28
IDUA	iduronidase, alpha-L-	4p16.3
IFT122	intraflagellar transport 122	3q21
IFT140	intraflagellar transport 140	16p13.3
IFT172	intraflagellar transport 172	2p23.3
IFT27	intraflagellar transport 27	22q13.1
IFT43	intraflagellar transport 43	14q24.3
IFT80	intraflagellar transport 80	3q25.33
IGBP1	immunoglobulin (CD79A) binding protein 1	Xq13.1-q13.3
IGF1R	insulin-like growth factor 1 receptor	15q26.3
IHH	indian hedgehog	2q33-q35
IKBKG	inhibitor of kappa light polypeptide gene enhancer in B-cells, kinase gamma	Xq28
IL11RA	interleukin 11 receptor, alpha	9p13
IL17RD	interleukin 17 receptor D	3p21.1
IL1RAPL1	interleukin 1 receptor accessory protein-like 1	Xp22.1-p21.3
IL21	interleukin 21	4q26-q27
IMPAD1	inositol monophosphatase domain containing 1	8q12.1
INHBA	inhibin, beta A	7p15-p13
INPP5E	inositol polyphosphate-5-phosphatase, 72 kDa	9q34.3
INPPL1	inositol polyphosphate phosphatase-like 1	11q23
INSR	insulin receptor	19p13.3-p13.2
IQSEC2	IQ motif and Sec7 domain 2	Xp11.23
IRF6	interferon regulatory factor 6	1q32.2-q32.3
IRX5	iroquois homeobox 5	16q12.2
ITCH	itchy E3 ubiquitin protein ligase	20q11.22
ITGB3	integrin, beta 3 (platelet glycoprotein IIIa, antigen CD61)	17q21.32
JAG1	jagged 1	20p12.1-p11.23
JAG2	jagged 2	14q32

KAL1	Kallmann syndrome 1 sequence	Xp22.32
KANSL1	KAT8 regulatory NSL complex subunit 1	17q21.31
KAT6B	K(lysine) acetyltransferase 6B	10q22.2
KCNJ11	potassium inwardly-rectifying channel, subfamily J, member 11	11p15.1
KCNJ13	potassium inwardly-rectifying channel, subfamily J, member 13	2q37
KCNJ2	potassium inwardly-rectifying channel, subfamily J, member 2	17q24.3
KCNK9	potassium channel, subfamily K, member 9	8q24.3
KCNQ1OT1	KCNQ1 opposite strand/antisense transcript 1 (non-protein coding)	11p15.5
KCNV2	potassium channel, subfamily V, member 2	9p24.2
KCTD1	potassium channel tetramerization domain containing 1	18q11.2
KDM6A	lysine (K)-specific demethylase 6A	Xp11.2
KIAA0196	KIAA0196	8q24.13
KIAA1279	KIAA1279	10q22.1
KIF11	kinesin family member 11	10q24.1
KIF22	kinesin family member 22	16p11.2
KIF7	kinesin family member 7	15q26.1
KIRREL3	kin of IRRE like 3 (Drosophila)	11q24
KISS1	KiSS-1 metastasis-suppressor	1q32
KISS1R	KISS1 receptor	19p13.3
KIT	v-kit Hardy-Zuckerman 4 feline sarcoma viral oncogene homolog	4q12
KITLG	KIT ligand	12q22
KL	klotho	13q12
KLHL41	kelch-like family member 41	2q31.1
KLHL7	kelch-like family member 7	7p15.3
KLK1	kallikrein 1	19q13.3
KMT2A	lysine (K)-specific methyltransferase 2A	11q23
KMT2D	lysine (K)-specific methyltransferase 2D	12q13.12
KRAS	Kirsten rat sarcoma viral oncogene homolog	12p12.1
KRIT1	KRIT1, ankyrin repeat containing	7q21.2
KRT1	keratin 1	12q13.13

KRT10	keratin 10	17q21.2
KRT14	keratin 14	17q21.2
KRT5	keratin 5	12q13.13
KRT9	keratin 9	17q21.2
L1CAM	L1 cell adhesion molecule	Xq28
LAMB2	laminin, beta 2 (laminin S)	3p21.3-p21.2
LHX8	LIM homeobox 8	1p31.1
LIPH	lipase, member H	3q27
LMBR1	limb development membrane protein 1	7q36.3
LMNA	lamin A/C	1q22
LMX1B	LIM homeobox transcription factor 1, beta	9q33.3
LRBA	LPS-responsive vesicle trafficking, beach and anchor containing	4q13
LRP4	low density lipoprotein receptor-related protein 4	11p11.2
LRP8	low density lipoprotein receptor-related protein 8, apolipoprotein e receptor	1p32.3
LRPPRC	leucine-rich pentatricopeptide repeat containing	2p21
LTBP2	latent transforming growth factor beta binding protein 2	14q24.3
LTBP4	latent transforming growth factor beta binding protein 4	19q13.1-q13.2
LYST	lysosomal trafficking regulator	1q42.1-q42.2
LZTFL1	leucine zipper transcription factor-like 1	3p21.3
MAB21L2	mab-21-like 2 (C. elegans)	4q31.3
MAFB	v-maf avian musculoaponeurotic fibrosarcoma oncogene homolog B	20q11.1-q13.1
MALT1	mucosa associated lymphoid tissue lymphoma translocation gene 1	18q21
MAP2	microtubule-associated protein 2	2q34-q35
MAP2K1	mitogen-activated protein kinase kinase 1	15q22.1-q22.33
MAP2K2	mitogen-activated protein kinase kinase 2	19p13.3
MAPT	microtubule-associated protein tau	17q21
MASP1	mannan-binding lectin serine peptidase 1 (C4/C2 activating component of Ra-reactive factor)	3q27-q28
MBD5	methyl-CpG binding domain protein 5	2q23.2
MBOAT1	membrane bound O-acyltransferase domain containing 1	6p22.3
MBS1	Moebius syndrome 1	13q12.2

MBTPS2	membrane-bound transcription factor peptidase, site 2	Xp22.12-p22.11
MC2R	melanocortin 2 receptor (adrenocorticotropic hormone)	18p11.2
MCM4	minichromosome maintenance complex component 4	8q12-q13
MCPH1	microcephalin 1	8p23.1
MECP2	methyl CpG binding protein 2	Xq28
MED12	mediator complex subunit 12	Xq13
MEF2C	myocyte enhancer factor 2C	5q14.3
MEGF10	multiple EGF-like-domains 10	5q33
MEGF8	multiple EGF-like-domains 8	19q13.2
MEIS2	Meis homeobox 2	15q14
MELK	maternal embryonic leucine zipper kinase	9p13.1
MEN1	multiple endocrine neoplasia I	11q13
MEOX1	mesenchyme homeobox 1	17q21.31
MEOX2	mesenchyme homeobox 2	7p22.1-p21.3
MID1	midline 1	Xp22
MIPOL1	mirror-image polydactyly 1	14q13.3
MIR140	microRNA 140	16q22.1
MIR17HG	miR-17-92 cluster host gene (non-protein coding)	13q31.3
MKKS	McKusick-Kaufman syndrome	20p12
MKS1	Meckel syndrome, type 1	17q21-q24
MKX	mohawk homeobox	10p12.1
MLH1	mutL homolog 1	3p22.3
MMP2	matrix metallopeptidase 2 (gelatinase A, 72kDa gelatinase, 72kDa type IV collagenase)	16q13-q21
MOGS	mannosyl-oligosaccharide glucosidase	2p13.1
MPP7	membrane protein, palmitoylated 7 (MAGUK p55 subfamily member 7)	10p12.1
MRPS16	mitochondrial ribosomal protein S16	10q22.1
MRXS11	mental retardation, X-linked, syndromic 11	Xq26-q27
MSX1	msh homeobox 1	4p16.2
MSX2	msh homeobox 2	5q35.2
MTHFR	methylenetetrahydrofolate reductase (NAD(P)H)	1p36.3

MTND3P1	MT-ND3 pseudogene 1	13q12.11
MTR	5-methyltetrahydrofolate-homocysteine methyltransferase	1q43
MUC5B	mucin 5B, oligomeric mucus/gel-forming	11p15.5
MYCN	v-myc avian myelocytomatosis viral oncogene neuroblastoma derived homolog	2p24.3
MYH3	myosin, heavy chain 3, skeletal muscle, embryonic	17p13.1
MYH8	myosin, heavy chain 8, skeletal muscle, perinatal	17p13.1
MYMY1	moyamoya disease 1	3p26-p24.2
NAA10	N(alpha)-acetyltransferase 10, NatA catalytic subunit	Xq28
NALCN	sodium leak channel, non-selective	13q32.3
NBAS	neuroblastoma amplified sequence	2p24.3
NBN	nibrin	8q21-q24
NDN	necdin, melanoma antigen (MAGE) family member	15q11-q12
NDUFAF2	NADH dehydrogenase (ubiquinone) complex I, assembly factor 2	5q12.1
NEB	nebulin	2q22
NEBL	nebulette	10p12
NEK1	NIMA-related kinase 1	4q32.3
NF1	neurofibromin 1	17q11.2
NFATC2	nuclear factor of activated T-cells, cytoplasmic, calcineurin-dependent 2	20q13.2
NFIA	nuclear factor I/A	1p31.3-p31.2
NFIX	nuclear factor I/X (CCAAT-binding transcription factor)	19p13.3
NFKB1	nuclear factor of kappa light polypeptide gene enhancer in B-cells 1	4q24
NFKB2	nuclear factor of kappa light polypeptide gene enhancer in B-cells 2 (p49/p100)	10q24
NHEJ1	nonhomologous end-joining factor 1	2q35
NHS	Nance-Horan syndrome (congenital cataracts and dental anomalies)	Xp22.3-p21.1
NIPAL4	NIPA-like domain containing 4	5q33.3
NIPBL	Nipped-B homolog (Drosophila)	5p13.2
NKX3-2	NK3 homeobox 2	4p16.3
NNT	nicotinamide nucleotide transhydrogenase	5p12
NOG	noggin	17q22
NOTCH2	notch 2	1p13-p11

NPAS4	neuronal PAS domain protein 4	11q13.2
NPHS2	nephrosis 2, idiopathic, steroid-resistant (podocin)	1q25-q31
NPR2	natriuretic peptide receptor 2	9p21-p12
NR0B1	nuclear receptor subfamily 0, group B, member 1	Xp21.3
NR2F1	nuclear receptor subfamily 2, group F, member 1	5q14
NRAS	neuroblastoma RAS viral (v-ras) oncogene homolog	1p13.2
NRN1	neuritin 1	6p25.1
NRXN1	neurexin 1	2p16.3
NSD1	nuclear receptor binding SET domain protein 1	5q35
NSDHL	NAD(P) dependent steroid dehydrogenase-like	Xq28
NSMF	NMDA receptor synaptonuclear signaling and neuronal migration factor	9q34.3
OASD	ocular albinism and sensorineural deafness	Xp22.3-p22.2
OCA2	oculocutaneous albinism II	15q12
OCLN	occludin	5q13.1
OFC1	orofacial cleft 1	6p24
OFCC1	orofacial cleft 1 candidate 1	6p24.3
OFD1	oral-facial-digital syndrome 1	Xp22
OPHN1	oligophrenin 1	Xq12
ORC1	origin recognition complex, subunit 1	1p32
ORC4	origin recognition complex, subunit 4	2q22-q23
OSR2	odd-skipped related transciption factor 2	8q22.2
OTX2	orthodenticle homeobox 2	14q22.3
PACS1	phosphofurin acidic cluster sorting protein 1	11q13.1-q13.2
PAFAH1B1	platelet-activating factor acetylhydrolase 1b, regulatory subunit 1 (45kDa)	17p13.3
PAK3	p21 protein (Cdc42/Rac)-activated kinase 3	Xq22.3
PALB2	partner and localizer of BRCA2	16p12.1
PAPSS2	3'-phosphoadenosine 5'-phosphosulfate synthase 2	10q24
PAX1	paired box 1	20p11.22
PAX2	paired box 2	10q24.31
PAX3	paired box 3	2q36.1

PAX6	paired box 6	11p13
PAX7	paired box 7	1p36.13
PAX9	paired box 9	14q13.3
PCNT	pericentrin	21q22.3
PCYT1A	phosphate cytidylyltransferase 1, choline, alpha	3q29
PDE4D	phosphodiesterase 4D, cAMP-specific	5q12
PDE6D	phosphodiesterase 6D, cGMP-specific, rod, delta	2q35-q36
PDGFC	platelet derived growth factor C	4q32
PDGFRA	platelet-derived growth factor receptor, alpha polypeptide	4q12
PDHA1	pyruvate dehydrogenase (lipoamide) alpha 1	Xp22.1
PDR	pigment disorder, reticulate	Xp22-p21
PEPD	peptidase D	19q13.11
PEX1	peroxisomal biogenesis factor 1	7q21.2
PEX10	peroxisomal biogenesis factor 10	1p36.32
PEX12	peroxisomal biogenesis factor 12	17q21.1
PEX13	peroxisomal biogenesis factor 13	2p16.1
PEX14	peroxisomal biogenesis factor 14	1p36.22
PEX16	peroxisomal biogenesis factor 16	11p
PEX19	peroxisomal biogenesis factor 19	1q22
PEX2	peroxisomal biogenesis factor 2	8q21.11
PEX26	peroxisomal biogenesis factor 26	22q11.21
PEX3	peroxisomal biogenesis factor 3	6q24.2
PEX5	peroxisomal biogenesis factor 5	12p
PEX6	peroxisomal biogenesis factor 6	6p22-p11
PEX7	peroxisomal biogenesis factor 7	6q21-q22.2
PGAP2	post-GPI attachment to proteins 2	11p15.4
PGAP3	post-GPI attachment to proteins 3	17q21.2
PGK1	phosphoglycerate kinase 1	Xq13.3
PGM1	phosphoglucomutase 1	1p22.1
PHF21A	PHD finger protein 21A	11p11.2

PHF8	PHD finger protein 8	Xp11.22
PHGDH	phosphoglycerate dehydrogenase	1p12
PIEZO2	piezo-type mechanosensitive ion channel component 2	18p11.21
PIGA	phosphatidylinositol glycan anchor biosynthesis, class A	Xp22.1
PIGL	phosphatidylinositol glycan anchor biosynthesis, class L	17p12-p11.2
PIGN	phosphatidylinositol glycan anchor biosynthesis, class N	18q21.33
PIGV	phosphatidylinositol glycan anchor biosynthesis, class V	1p36.11
PIK3CA	phosphatidylinositol-4,5-bisphosphate 3-kinase, catalytic subunit alpha	3q26.3
PIK3R1	phosphoinositide-3-kinase, regulatory subunit 1 (alpha)	5q13.1
PIK3R2	phosphoinositide-3-kinase, regulatory subunit 2 (beta)	19p13.11
PITX1	paired-like homeodomain 1	5q31.1
PITX2	paired-like homeodomain 2	4q25
PKD1	polycystic kidney disease 1 (autosomal dominant)	16p13.3
PKD2	polycystic kidney disease 2 (autosomal dominant)	4q22.1
PKHD1	polycystic kidney and hepatic disease 1 (autosomal recessive)	6p21.2-p12
PKLR	pyruvate kinase, liver and RBC	1q22
PLCB4	phospholipase C, beta 4	20p12
PLEC	plectin	8q24
PLOD1	procollagen-lysine, 2-oxoglutarate 5-dioxygenase 1	1p36.22
PLOD3	procollagen-lysine, 2-oxoglutarate 5-dioxygenase 3	7q22.1
PMM2	phosphomannomutase 2	16p13
POC1A	POC1 centriolar protein A	3p21.2
POFUT1	protein O-fucosyltransferase 1	20q11
POGLUT1	protein O-glucosyltransferase 1	3q13.33
POLE	polymerase (DNA directed), epsilon, catalytic subunit	12q24.3
POLG	polymerase (DNA directed), gamma	15q24
POLR1C	polymerase (RNA) I polypeptide C, 30kDa	6p21.1
POLR1D	polymerase (RNA) I polypeptide D, 16kDa	13q12.2
POMGNT1	protein O-linked mannose N-acetylglucosaminyltransferase 1 (beta 1,2-)	1p34.1
POMT1	protein-O-mannosyltransferase 1	9q34.1

POMT2	protein-O-mannosyltransferase 2	14q24
PORCN	porcupine homolog (Drosophila)	Xp11.23
PPOX	protoporphyrinogen oxidase	1q22
PQBP1	polyglutamine binding protein 1	Xp11.23
PRDM16	PR domain containing 16	1p36.23-p33
PREPL	prolyl endopeptidase-like	2p22.1
PRICKLE1	prickle homolog 1 (Drosophila)	12p11-q12
PRKAR1A	protein kinase, cAMP-dependent, regulatory, type I, alpha	17q24.2
PROKR2	prokineticin receptor 2	20p12.3
PRPH2	peripherin 2 (retinal degeneration, slow)	6p21.1
PRRX1	paired related homeobox 1	1q24.3
PRSS12	protease, serine, 12 (neurotrypsin, motopsin)	4q25-q26
PTCH1	patched 1	9q22.1-q31
PTCH2	patched 2	1p34.1
PTDSS1	phosphatidylserine synthase 1	8q22
PTEN	phosphatase and tensin homolog	10q23
PTH1R	parathyroid hormone 1 receptor	3p22-p21.1
PTHLH	parathyroid hormone-like hormone	12p12.1-p11.2
PTPN11	protein tyrosine phosphatase, non-receptor type 11	12q24.1
PUF60	poly-U binding splicing factor 60KDa	8q24.3
PUS1	pseudouridylate synthase 1	12q24
PVRL1	poliovirus receptor-related 1 (herpesvirus entry mediator C)	11q23-q24
PVRL4	poliovirus receptor-related 4	1q22-q23.2
PYCR1	pyrroline-5-carboxylate reductase 1	17q25.3
RAB23	RAB23, member RAS oncogene family	6p12.1
RAB28	RAB28, member RAS oncogene family	4p16.1
RAB3GAP1	RAB3 GTPase activating protein subunit 1 (catalytic)	2q21.3
RAD21	RAD21 homolog (S. pombe)	8q24.11
RAF1	Raf-1 proto-oncogene, serine/threonine kinase	3p25
RAI1	retinoic acid induced 1	17p11.2

RAPSN	receptor-associated protein of the synapse	11p11.2
RARB	retinoic acid receptor, beta	3p24
RB1	retinoblastoma 1	13q14.2
RBBP8	retinoblastoma binding protein 8	18q11.2
RBFOX1	RNA binding protein, fox-1 homolog (C. elegans) 1	16p13.3
RBM10	RNA binding motif protein 10	Xp11.3
RBM28	RNA binding motif protein 28	7q32.2
RBPJ	recombination signal binding protein for immunoglobulin kappa J region	4p15.2
RDH12	retinol dehydrogenase 12 (all-trans/9-cis/11-cis)	14q24.1
RECQL4	RecQ protein-like 4	8q24.3
RELA	v-rel avian reticuloendotheliosis viral oncogene homolog A	11q13
RELN	reelin	7q22
RFX3	regulatory factor X, 3 (influences HLA class II expression)	9p24.2
RIEG2	Rieger syndrome 2	13q14
RIN2	Ras and Rab interactor 2	20p11.22
RIPK4	receptor-interacting serine-threonine kinase 4	21q22.3
RIT1	Ras-like without CAAX 1	1q21.2
RMRP	RNA component of mitochondrial RNA processing endoribonuclease	9p21-p12
RNF135	ring finger protein 135	17q11.2
RNU4ATAC	RNA, U4atac small nuclear (U12-dependent splicing)	2q14.2
ROGDI	rogdi homolog (Drosophila)	16p13.3
ROR2	receptor tyrosine kinase-like orphan receptor 2	9q22
RPGRIP1L	RPGRIP1-like	16q12.2
RPL11	ribosomal protein L11	1p36.1-p35
RPL15	ribosomal protein L15	3p24.1
RPL26	ribosomal protein L26	17p13
RPL35A	ribosomal protein L35a	3q29
RPL36	ribosomal protein L36	19p13.2
RPL5	ribosomal protein L5	1p22.1
RPS10	ribosomal protein S10	6p21.31

RPS15	ribosomal protein S15	19p13.3
RPS17	ribosomal protein S17	15q25.2
RPS19	ribosomal protein S19	19q13.2
RPS24	ribosomal protein S24	10q22
RPS26	ribosomal protein S26	12q13
RPS27A	ribosomal protein S27a	2p16
RPS6KA3	ribosomal protein S6 kinase, 90kDa, polypeptide 3	Xp22.2-p22.1
RPS7	ribosomal protein S7	2p25
RREB1	ras responsive element binding protein 1	6p25
RUNX2	runt-related transcription factor 2	6p21
RYK	receptor-like tyrosine kinase	3q22.1
RYR1	ryanodine receptor 1 (skeletal)	19q13.1
SALL1	spalt-like transcription factor 1	16q12.1
SALL4	spalt-like transcription factor 4	20q13.2
SATB2	SATB homeobox 2	2q33.1
SC5D	sterol-C5-desaturase	11q23.3
SCARF2	scavenger receptor class F, member 2	22q11.21
SCD5	stearoyl-CoA desaturase 5	4q21.3
SCLT1	sodium channel and clathrin linker 1	4q28.2
SCN1A	sodium channel, voltage-gated, type I, alpha subunit	2q24.3
SCN2A	sodium channel, voltage-gated, type II, alpha subunit	2q24.3
SCRIB	scribbled planar cell polarity protein	8q24.3
SCZD1	schizophrenia disorder 1	5q11.2-q13.3
SEC23A	Sec23 homolog A (S. cerevisiae)	14q21.1
SEMA3A	sema domain, immunoglobulin domain (Ig), short basic domain, secreted, (semaphorin) 3A	7p12.1
SEMA3E	sema domain, immunoglobulin domain (Ig), short basic domain, secreted, (semaphorin) 3E	7q21.11
SEPN1	selenoprotein N, 1	1p36.13
SEPT9	septin 9	17q25.3
SERPINC1	serpin peptidase inhibitor, clade C (antithrombin), member 1	1q25.1
SERPINH1	serpin peptidase inhibitor, clade H (heat shock protein 47), member 1, (collagen binding protein 1)	11q13.5

SETBP1	SET binding protein 1	18q21.1
SETD5	SET domain containing 5	3p25.3
SF3B4	splicing factor 3b, subunit 4, 49kDa	1q21.2
SFTPA1	surfactant protein A1	10q22.3
SFTPA2	surfactant protein A2	10q22.3
SFTPB	surfactant protein B	2p12-p11.2
SFTPC	surfactant protein C	8p21
SH3BP2	SH3-domain binding protein 2	4p16.3
SH3PXD2B	SH3 and PX domains 2B	5q35.2
SHANK3	SH3 and multiple ankyrin repeat domains 3	22q13.3
SHFM1	split hand/foot malformation (ectrodactyly) type 1	7q21.3
SHFM2	split hand/foot malformation (ectrodactyly) type 2	Xq26
SHFM5	split hand/foot malformation (ectrodactyly) type 5	2q31
SHH	sonic hedgehog	7q36
SHOX	short stature homeobox	Xp22.33 and Yp11.32
SIL1	SIL1 nucleotide exchange factor	5q31
SIX3	SIX homeobox 3	2p21
SIX6	SIX homeobox 6	14q23.1
SKI	SKI proto-oncogene	1p36.33
SLC12A6	solute carrier family 12 (potassium/chloride transporter), member 6	15q13
SLC19A1	solute carrier family 19 (folate transporter), member 1	21q22.3
SLC1A3	solute carrier family 1 (glial high affinity glutamate transporter), member 3	5p13
SLC25A1	solute carrier family 25 (mitochondrial carrier; citrate transporter), member 1	22q11
SLC26A2	solute carrier family 26 (anion exchanger), member 2	5q31-q34
SLC29A3	solute carrier family 29 (equilibrative nucleoside transporter), member 3	10q22.2
SLC2A10	solute carrier family 2 (facilitated glucose transporter), member 10	20q13.12
SLC38A8	solute carrier family 38, member 8	16q23.3
SLC6A8	solute carrier family 6 (neurotransmitter transporter), member 8	Xq28
SLC7A9	solute carrier family 7 (amino acid transporter light chain, bo,+ system), member 9	19q13.11
SLC9A6	solute carrier family 9, subfamily A (NHE6, cation proton antiporter 6), member 6	Xq26.3

SLCO2A1	solute carrier organic anion transporter family, member 2A1	3q21
SLURP1	secreted LY6/PLAUR domain containing 1	8q24.3
SLX4	SLX4 structure-specific endonuclease subunit	16p13.3
SMAD2	SMAD family member 2	18q21
SMAD3	SMAD family member 3	15q21-q22
SMAD4	SMAD family member 4	18q21.1
SMARCA2	SWI/SNF related, matrix associated, actin dependent regulator of chromatin, subfamily a, member 2	9p24.3
SMARCAD1	SWI/SNF-related, matrix-associated actin-dependent regulator of chromatin, subfamily a, containing DEAD/H box 1	4q22-q23
SMARCB1	SWI/SNF related, matrix associated, actin dependent regulator of chromatin, subfamily b, member 1	22q11.23
SMC1A	structural maintenance of chromosomes 1A	Xp11.22-p11.21
SMC3	structural maintenance of chromosomes 3	10q25
SMOC1	SPARC related modular calcium binding 1	14q24.1
SMS	spermine synthase	Xp22.1
SNAI2	snail family zinc finger 2	8q11.21
SNAP29	synaptosomal-associated protein, 29kDa	22q11.21
SNIP1	Smad nuclear interacting protein 1	1p34.3
SNRPN	small nuclear ribonucleoprotein polypeptide N	15q11.2
SNTG1	syntrophin, gamma 1	8q11.21
SOS1	son of sevenless homolog 1 (Drosophila)	2p21
SOST	sclerostin	17q12-q21
SOX10	SRY (sex determining region Y)-box 10	22q13.1
SOX2	SRY (sex determining region Y)-box 2	3q26.3-q27
SOX3	SRY (sex determining region Y)-box 3	Xq27.1
SOX9	SRY (sex determining region Y)-box 9	17q24.3
SP8	Sp8 transcription factor	7p21.2
SPECC1L	sperm antigen with calponin homology and coiled-coil domains 1-like	22q11.23
SPG20	spastic paraplegia 20 (Troyer syndrome)	13q13.1
SPG23	spastic paraplegia 23 (autosomal recessive)	1q24-q32
SPINT2	serine peptidase inhibitor, Kunitz type, 2	19q13.2
SPRED1	sprouty-related, EVH1 domain containing 1	15q14

SPRY2	sprouty homolog 2 (Drosophila)	13q31.1
SPRY4	sprouty homolog 4 (Drosophila)	5q31.3
SPTAN1	spectrin, alpha, non-erythrocytic 1	9q34.11
SRCAP	Snf2-related CREBBP activator protein	16p11.2
SRY	sex determining region Y	Yp11.3
ST3GAL5	ST3 beta-galactoside alpha-2,3-sialyltransferase 5	2p11.2
ST5	suppression of tumorigenicity 5	11p15
STAC3	SH3 and cysteine rich domain 3	12q13.3
STAMBP	STAM binding protein	2p24.3-p24.1
STAR	steroidogenic acute regulatory protein	8p11.2
STK11	serine/threonine kinase 11	19p13.3
STRA6	stimulated by retinoic acid 6	15q24.1
STX16	syntaxin 16	20q13.32
STXBP1	syntaxin binding protein 1	9q34.1
SUCLA2	succinate-CoA ligase, ADP-forming, beta subunit	13q12.2-q13.3
SUFU	suppressor of fused homolog (Drosophila)	10q24.32
SUMO1	small ubiquitin-like modifier 1	2q33
SUOX	sulfite oxidase	12q13.13
SZT2	seizure threshold 2 homolog (mouse)	1p34.2
TACR3	tachykinin receptor 3	4q25
TALDO1	transaldolase 1	11p15.5-p15.4
TBC1D24	TBC1 domain family, member 24	16p13.3
TBC1D32	TBC1 domain family, member 32	6q22.31
TBCE	tubulin folding cofactor E	1q42.3
TBX1	T-box 1	22q11.21
TBX10	T-box 10	11q13.2
TBX15	T-box 15	1p11.1
TBX22	T-box 22	Xq21.1
TBX5	T-box 5	12q24.1
TCF12	transcription factor 12	15q21

TCF4	transcription factor 4	18q21.1
TCOF1	Treacher Collins-Franceschetti syndrome 1	5q32
TCTN2	tectonic family member 2	12q24.31
TCTN3	tectonic family member 3	10q24.1
TDGF1	teratocarcinoma-derived growth factor 1	3p21.31
TERC	telomerase RNA component	3q26.2
TFAP2A	transcription factor AP-2 alpha (activating enhancer binding protein 2 alpha)	6p24.3
TFAP2B	transcription factor AP-2 beta (activating enhancer binding protein 2 beta)	6p12
TFDP1	transcription factor Dp-1	13q34
TFR2	transferrin receptor 2	7q22
TGFA	transforming growth factor, alpha	2p13
TGFB1	transforming growth factor, beta 1	19q13.1
TGFB3	transforming growth factor, beta 3	14q24
TGFBR1	transforming growth factor, beta receptor 1	9q22
TGFBR2	transforming growth factor, beta receptor II (70/80kDa)	3p22
TGIF1	TGFB-induced factor homeobox 1	18p11.31
THADA	thyroid adenoma associated	2p21
THAP1	THAP domain containing, apoptosis associated protein 1	8p11.1
THAS	thoracoabdominal syndrome	X
THRB	thyroid hormone receptor, beta	3p24.2
TINF2	TERF1 (TRF1)-interacting nuclear factor 2	14q12
TMCO1	transmembrane and coiled-coil domains 1	1q22-q25
TMEM216	transmembrane protein 216	11q13.1
TMEM67	transmembrane protein 67	8q22.1
TNNI2	troponin I type 2 (skeletal, fast)	11p15.5
TNNT3	troponin T type 3 (skeletal, fast)	11p15.5
TOR1A	torsin family 1, member A (torsin A)	9q32-q34
TP63	tumor protein p63	3q27-q29
TPM1	tropomyosin 1 (alpha)	15q22.1
TPM2	tropomyosin 2 (beta)	9p13

TPM3	tropomyosin 3	1q21.2
TRAF6	TNF receptor-associated factor 6, E3 ubiquitin protein ligase	11p12
TRAPPC9	trafficking protein particle complex 9	8q24.3
TRIM32	tripartite motif containing 32	9q33.1
TRIM37	tripartite motif containing 37	17q
TRPS1	trichorhinophalangeal syndrome I	8q23.3
TRPV4	transient receptor potential cation channel, subfamily V, member 4	12q24.1
TSC2	tuberous sclerosis 2	16p13.3
TTC21B	tetratricopeptide repeat domain 21B	2q24.3
TTC8	tetratricopeptide repeat domain 8	14q31.3
TTI2	TELO2 interacting protein 2	8p12
TUBB2B	tubulin, beta 2B class IIb	6p25.2
TWIST1	twist family bHLH transcription factor 1	7p21
TWIST2	twist family bHLH transcription factor 2	2q37.3
TYMS	thymidylate synthetase	18p11.31-p11.21
UBA1	ubiquitin-like modifier activating enzyme 1	Xp11.23
UBB	ubiquitin B	17p12-p11.2
UBE3B	ubiquitin protein ligase E3B	12q24.12
UFD1L	ubiquitin fusion degradation 1 like (yeast)	22q11.2
UGT1A9	UDP glucuronosyltransferase 1 family, polypeptide A9	2q37
UROD	uroporphyrinogen decarboxylase	1p34
USB1	U6 snRNA biogenesis 1	16q13
VAX1	ventral anterior homeobox 1	10q26.11
VPS33B	vacuolar protein sorting 33 homolog B (yeast)	15q26.1
VSX1	visual system homeobox 1	20p11.21
WDPCP	WD repeat containing planar cell polarity effector	2p15
WDR11	WD repeat domain 11	10q26
WDR19	WD repeat domain 19	4p14
WDR34	WD repeat domain 34	9q34.11
WDR35	WD repeat domain 35	2p24.3

WDR60	WD repeat domain 60	7q36.3
WDR62	WD repeat domain 62	19q13.12
WNK1	WNK lysine deficient protein kinase 1	12p13.3
WNT10B	wingless-type MMTV integration site family, member 10B	12q13
WNT3	wingless-type MMTV integration site family, member 3	17q21-q22
WNT4	wingless-type MMTV integration site family, member 4	1p36.23-p35.1
WNT5A	wingless-type MMTV integration site family, member 5A	3p21-p14
WNT7A	wingless-type MMTV integration site family, member 7A	3p25
WNT9B	wingless-type MMTV integration site family, member 9B	17q21
WT1	Wilms tumor 1	11p13
XIST	X inactive specific transcript (non-protein coding)	Xq13.2
XPC	xeroderma pigmentosum, complementation group C	3p25.1
YAP1	Yes-associated protein 1	11q13
YPEL1	yippee-like 1 (Drosophila)	22q11.2
YWHAE	tyrosine 3-monooxygenase/tryptophan 5-monooxygenase activation protein, epsilon	17p13.3
ZBTB16	zinc finger and BTB domain containing 16	11q23
ZBTB24	zinc finger and BTB domain containing 24	6q21
ZC4H2	zinc finger, C4H2 domain containing	Xq11.1
ZEB2	zinc finger E-box binding homeobox 2	2q22.3
ZFHX4	zinc finger homeobox 4	8q21.11
ZFP37	ZFP37 zinc finger protein	9q32
ZFP57	ZFP57 zinc finger protein	6p22.1
ZFP90	ZFP90 zinc finger protein	16q22.1
ZIC2	Zic family member 2	13q32
ZIC3	Zic family member 3	Xq24-q27.1
ZMPSTE24	zinc metallopeptidase STE24	1p34
ZNF335	zinc finger protein 335	20q13.12
ZNF81	zinc finger protein 81	Xp11.23
